# The Electrical and Mechanical Characteristics of Conductive PVA/PEDOT:PSS Hydrogel Foams for Soft Strain Sensors

**DOI:** 10.3390/s24020570

**Published:** 2024-01-16

**Authors:** Florian E. Jurin, Cédric C. Buron, Eleonora Frau, Stefan del Rossi, Silvia Schintke

**Affiliations:** 1 Institut UTINAM, UMR 6213 CNRS-UBFC, Université de Bourgogne Franche-Comté (UBFC), F-25030 Besançon Cedex, France; cedric.buron@univ-fcomte.fr; 2 Laboratory of Applied NanoSciences (COMATEC-LANS), University of Applied Sciences Western Switzerland (HES-SO), CH-1401 Yverdon-les-Bains, Switzerland

**Keywords:** hydrogel, foam, resistive strain sensors, electrical broadband spectroscopy, conductivity, surfactant

## Abstract

Conductive hydrogels are of interest for highly flexible sensor elements. We compare conductive hydrogels and hydrogel foams in view of strain-sensing applications. Polyvinyl alcool (PVA) and poly(3,4-ethylenedioxythiophene (PEDOT:PSS) are used for the formulation of conductive hydrogels. For hydrogel foaming, we have investigated the influence of dodecylbenzenesulfonate (DBSA) as foaming agent, as well as the influence of air incorporation at various mixing speeds. We showed that DBSA acting as a surfactant, already at a concentration of 1.12wt%, efficiently stabilizes air bubbles, allowing for the formulation of conductive PVA and PVA/PEDOT:PSS hydrogel foams with low density (<400 kg/m^3^) and high water uptake capacity (swelling ratio > 1500%). The resulting Young moduli depend on the air-bubble incorporation from mixing, and are affected by freeze-drying/rehydration. Using dielectric broadband spectroscopy under mechanical load, we demonstrate that PVA/PEDOT:PSS hydrogel foams exhibit a significant decrease in conductivity under mechanical compression, compared to dense hydrogels. The frequency-dependent conductivity of the hydrogels exhibits two plateaus, one in the low frequency range, and one in the high frequency range. We find that the conductivity of the PVA/PEDOT:PSS hydrogels decreases linearly as a function of pressure in each of the frequency regions, which makes the hydrogel foams highly interesting in view of compressive strain-sensing applications.

## 1. Introduction

Advances in flexible conductive polymer materials have seen remarkable growth in recent years, opening avenues for their integration into electronic devices. Strain sensors, essential in the field of flexible electronics, play a transformative role in capturing and interpreting mechanical deformations. Such sensors, designed to perceive changes in shape, compression or elongation, convert physical changes into measurable electrical signals. With applications ranging from wearable technology to structural health monitoring, strain sensors are contributing to advances in robotics, healthcare and materials science [1,2,3,4]. Their ability to detect and quantify mechanical stresses provides data to optimize the performance, safety and efficiency of various systems, making them necessary tools in the field of reactive and adaptive technologies. The development of flexible strain sensors is closely linked to fabrication the techniques of flexible conductive polymer materials. Notable approaches include, among others, the chemical or electrochemical polymerization of conductive polymers [5,6], the creation of multilayer polyelectrolyte films by layer-by-layer method [7,8], as well as conducting soft polymer composites [9,10]. An emerging field in this context is that of conductive hydrogels [11,12,13,14,15].

Hydrogels, solid materials with 3D cross-linked hydrophilic polymer networks, have exceptional characteristics that set them apart. Their network structures, coupled with strong hydrophilicity, allow for significant water absorption without dissolution. These materials exhibit a multitude of qualities, including biocompatibility, flexibility, high extensibility, high transparency, and high viscoelasticity. Some hydrogels exhibit even self-adhesion when using specific polymers [16,17]. These properties position hydrogels as interesting candidates for the fabrication of flexible strain sensors. Various polymers can be used to make hydrogels, such as polyacrylic acid (PAA), polyacrylamide (PAM), poly(2-hydroxyethyl methacrylate) (pHEMA), or polyvinyl alcohol (PVA) [18,19,20,21]. PVA, thanks to its -OH groups, appears to be a particularly promising candidate for the formulation of hydrogels, favoring cross-linked networks via intermolecular hydrogen bonds or chemical bonds [22,23]. The versatility of PVA hydrogels is further highlighted by their compatibility with various substances such as cross-linking agents (cellulose nanofibers, tannic acid, clay), readily adjusting mechanical properties [24,25].

In the formulation of conductive hydrogels, typically, conductive fillers such as graphene, carbon black, carbon nanotubes, metallic nanoparticles, and nanorods are incorporated into the hydrogel matrix [26,27]. Despite challenges like filler aggregation and inferior interfacial compatibility, conductive hydrogels with additional properties (optical, mechanical, chemical sensitivity) have been successfully developed. An alternative to conductive particles is the integration of conductive polymers, including polypyrrole, polyaniline, polythiophene, and poly(3,4-ethylenedioxythiophene) (PEDOT:PSS) [28,29,30]. PEDOT:PSS, as a leading electroconductive polymer, offers high electrical conductivity, aqueous processability, chemical stability, and cytocompatibility, making it widely applicable in sensor-based hydrogels [31,32,33].

By using conductive hydrogels, it is possible to produce self-supported substrates endowed with interesting mechanical properties, including resistance under tensile deformation, good flexibility, and self-healing properties [2]. The elaboration of flexible strain sensors using conductive hydrogels is achievable through diverse methods, such as utilizing molds of varying shapes or employing cutting-edge 3D printing technology [34,35,36]. The often-overlooked porosity, a result of the freeze-drying process [37,38], significantly influences the material’s overall characteristics.

Porosity can be generated by various fabrication techniques such as freeze-drying, the acid-induced decomposition of a foaming agent, and the porosigen technique, alongside the use of foam stabilizers [39,40,41]. Hydrogels featuring a porous composition are, e.g., of interest for increased user comfort in the field of wearable sensors. Hydrogels with a porous composition offer several advantages for strain-sensing applications such as a modification of their compressive strength. The porous network allows for a more even distribution of internal stress, contributing to improved mechanical performance [42]. Furthermore, the increased surface area of highly porous structures fosters enhanced interactions with the external environment, and is particularly advantageous for detection applications. These characteristics make porous hydrogels a preferred choice for developing effective and responsive strain sensors. The versatility of porous hydrogels extends beyond conventional applications, and includes, e.g., decontamination [43], flame resistance [44], tissue engineering [45] and sensing applications [46].

In this article, the development of porous conductive hydrogels, particularly in the form of hydrogel foams, is revealed, and their potential as robust strain sensing elements is explored. Based on PVA and PVA/PEDOT:PSS hydrogels, the impact of a foaming agent—dodecylbenzene sulfonate (DBSA)—in hydrogel formulation is further explored by our investigation. The influence of different mixing speeds to obtain hydrogels with pronounced porosity is also studied. The formulation of PVA/PEDOT:PSS hydrogel foams, characterized by low density and remarkable swelling properties, is demonstrated, and their feasibility is established. Through comprehensive mechanical and electrical characterization, our results confirm the attractiveness of conductive PVA/PEDOT:PSS hydrogel foams as superior candidates for strain sensors, particularly compared to gel-state hydrogels, in a wide range of frequencies.

## 2. Materials and Methods

### 2.1. Materials

For hydrogel formulation, polyvinyl alcohol (Mw 130,000 g/mol, Mowiol(R)18-88), glutaraldehyde solution (GA) (25%) as crosslinker agent, and sodium dodecylbenzenesulfonate (DBSA) as foam stabilizing additive were purchased from Sigma-Aldrich (Darmstadt, Germany). As conductive additive, poly(3,4-ethylenedioxythiophene) polystyrene sulfonate (PEDOT:PSS) aqueous solution (Clevios PH 1000, purchased from Heraeus, Hanau, Germany ) was used; the PEDOT:PSS solution exhibits a solid content of 1.3wt% and a PEDOT:PSS weight ratio of 1:2.5. The other chemicals were obtained from Sigma-Aldrich and used without further purification.

### 2.2. Formulation of Hydrogels

For the formulation of hydrogels, PVA solid was first added into deionized water (Milli-Q Plus, Millipore (Darmstadt, Germany), resistivity > 18 MΩ.cm) and dissolved by magnetic stirring at 95 °C for 4 h, until PVA was completely dissolved, resulting in a uniform PVA solution. PVA solutions were then mixed with PEDOT:PSS and magnetically stirred for 1 h at room temperature to form a PVA/PEDOT:PSS solution with a PVA/PEDOT:PSS ratio of 8:0.28 *w*/*w*.

For the formulation of dense hydrogels, various solutions with specific concentrations of DBSA (corresponding to 0wt%, 1.12wt% or 2.24wt%) were prepared by adding DBSA, as purchased, into PVA/PEDOT:PSS solutions, using magnetic stirring at 300 rpm for 5 min. For the gelation, 100 µL of GA of 12.5wt% concentration were added into the previous PVA/PEDOT:PSS solutions (with and without DBSA) and magnetically stirred (300 rpm, 1 min). The low mixing speed avoids the trapping of air during mixing, in order to obtain dense gels.

For the formulation of hydrogel foams, various DBSA/PVA/PEDOT:PSS solutions (20 mL in total) (same DBSA concentrations used as above) were poured into a beaker (4 cm of diameter × 8 cm height), and mixed for 4 min. During mixing, air was incorporated into the various solutions by mixing them at high speed. Two different dispersers were used for this purpose, an IKA Eurostar disperser with a 4-blade propeller (diameter: 3 cm) (used at 1000 rpm or 2000 rpm) and an IKA Ultra-Turrax T25-S25N 25F (used at 24,000 rpm). Then, 100 µL GA (at 12.5wt%) was added, as described above, for the gelation of the PVA/PEDOT:PSS solutions (with and without DBSA), in order to obtain hydrogel foams. The mixing of GA was performed at 1000 rpm, 30 s, to well homogenize the crosslinker agent GA within the foam structure.

**Figure 1:**
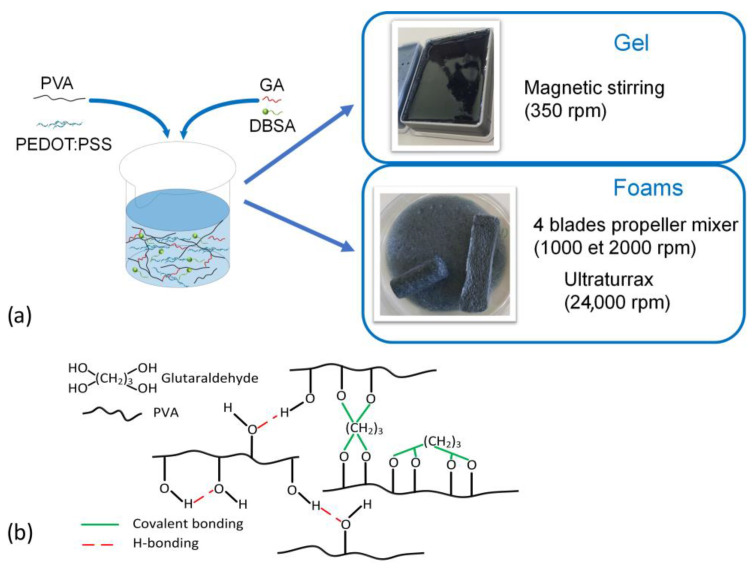
(**a**) Scheme showing the preparation process of PVA/PEDOT:PSS/DBSA hydrogels. (**b**) Schematic illustration of the different H-bonding and covalent bonding that can form the 3D network of hydrogel.

To facilitate the reading, hydrogel materials marked as GEL refer to dense hydrogels (prepared at 300 rpm), while hydrogel materials marked as F1, F2, or F24 refer to hydrogel foams (prepared at 1000 rpm or 2000 rpm, or 24,000 rpm, respectively). Furthermore, the preparation process of PVA/PEDOT:PSS hydrogels is graphically summarized (Figure 1a), together with schematics of the bonding mechanisms that are involved in the hydrogel network formation (Figure 1b).

Samples of the various hydrogels, i.e., gels and foams, were prepared by pouring the hydrogel solutions into Petri dishes (inner diameter, ID = 85 mm, and height, H = 10 mm) for their gelation at room temperature for 2 h.

For morphology analysis and for comparison, some of the PVA/PEDOT:PSS hydro-gels were frozen at −20 °C for 24 h and then freeze-dried for 24 h (freeze-dryer Christ—2-4 LSC Basic). Fresh hydrogel samples were stored at room temperature (22 ± 2 °C) in air under a controlled relative humidity of 80% in order to maintain their water content and avoid any drying out, while freeze-dried samples were rehydrated, prior to electrical and mechanical analysis.

### 2.3. The Characterization of Hydrogels

#### 2.3.1. Air Incorporation into Hydrogels: Density Analysis

Density analysis of the various hydrogels was performed using 5 mL plastic syringes. The syringes were weighed empty, then filled with the prepared hydrogel solution and weighed again after reticulation. The density of the obtained hydrogels is calculated using Equation (1):

(1) $$density=\frac{\left(m_{F}-m_{E}\right)}{V_{T}}$$

where *V_T_* is the total volume of hydrogel filled into the syringe, *m_F_* and *m_E_* are the mass of the filled and empty syringe, respectively. We note that the total volume upon gelation corresponds well to the initial volume filled into the syringe. For each of the various solutions, at least three syringes were filled with hydrogel for the analysis. The reported density values are average values, including their standard deviations, from triplicate experiments.

#### 2.3.2. Morphology Characterization

For morphology analysis, freeze-dried hydrogels were investigated using a scanning electron microscope (SEM) (TM1000, Hitachi, Tokyo, Japan) at a voltage of 15 kV. Conductive adhesive tape was used for fixing small sample pieces onto the SEM sample holder.

#### 2.3.3. The Water Uptake of Hydrogels: Swelling Ratio Analysis

To determine the water uptake capacity of the hydrogels, the swelling ratio (SR) of the samples was analyzed by immersing freeze-dried samples in deionized water, following the procedure described by Lee et al. [41]. The samples were shortly removed from the water for measuring their mass at several time intervals. For weighing, surface water was removed using blotting paper. The water uptake or swelling ratio, *SR*(*t*), measured as a function of time, is calculated using Equation (2):

(2) $$SR\left(t\right)=\frac{m\left(t\right)-m_{i}}{m_{i}}\times 100$$

where *m_i_* is the initial mass of the freeze-dried hydrogel and *m(t)* the mass of the hydrogel after a time t of rehydration.

#### 2.3.4. Rheological Analysis

A stress-controlled rheometer (Kinexus, Netzsch, Benton Harbor, MI, USA) equipped with circular parallel plates of 20 mm in diameter was used for the measurements. In order to measure the viscoelastic properties and gelation time by rheological analysis, hydrogel solutions were poured on the preheated/cooled rheometer plate. The upper part of the test geometry was lowered to the desired gap height (1 or 4 mm for gel and foam, respectively), and excess hydrogel was discarded. From the relative amplitude and phase of the oscillations, the elastic modulus (G′) and viscous modulus (G″) graphs were deduced.

The gelation time is determined as the crossover point between the elastic modulus (G′) and the viscous modulus (G″). The temperature was controlled by a Peltier system, measurements were performed at various temperatures between 5 °C and 50 °C. The gelation time is defined with respect to the moment of adding the crosslinker agent GA to the DBSA/PVA/PEDOT:PSS solutions. Five minutes were taken for sample handling prior to the recording of rheology data; therefore, 300 s are added to the read out of the crossover point of the elastic modulus (G′) and the viscous modulus (G″).

The overall viscoelastic properties of the hydrogels were evaluated using a dynamic strain sweep test sequence at an angular frequency of 1.0 Hz. Frequency sweep tests were conducted in the range of 0.1 Hz to 100 Hz, at a strain amplitude of 1.0% (in the linear viscoelastic region (LVR)). For determining the gelation time, measurements were then performed at 1.00 Hz with a strain amplitude of 1.0%.

#### 2.3.5. Electrical and Mechanical Characterization

The frequency-dependent electrical conductivity of hydrogels was investigated using broadband dielectric spectroscopy under various mechanical loads, with simultaneous analysis of the elastic modulus. Repeated cycling experiments of the electrical resistance were performed under various compressions by squeezing samples in the rheometer.

The electrical and mechanical behavior of disc-shaped hydrogels were analyzed for gels and foams, and compared for fresh and rehydrated samples. Detailed analysis has been performed for samples with 1.12wt% DBSA. Table 1 summarizes the hydrogel conditioning for electrical and mechanical characterization.

The frequency-dependent conductivity of hydrogels is characterized by broadband dielectric spectroscopy (frequency range between 0.01 Hz and 1.00 MHz) by applying an AC-voltage of 0.01 V and a DC-voltage of 0.00 V vs. Open Circuit Potential (OCP). Freeze-dried samples were rehydrated by immersing them in 250 mL of deionized water for 4 h, prior to analysis. Before each measurement, the OCP was measured over 300 s, in order to verify stable measurement conditions. The electrical characterization was carried out in a vertical measurement setup, i.e., by measuring vertically across a disc-shaped sample (area A, thickness d) sandwiched between two copper electrodes. The bottom electrode (diameter Ø_bottom_ = 5.0 cm) was used as counter and reference electrode, the top electrode (diameter Ø_top_ = 2.5 cm) as working electrode.

Dielectric spectra, in particular admittance spectra Y(ω), of the hydrogels were recorded for various compressive stress by applying a mechanical load, corresponding to a mass of up to 170 g, which is controlled by a load cell, when adjusting the distance between the electrodes using a micrometer screw.

#### 2.3.6. The Analysis of Electrical Conductivity and Young Modulus

In order to determine the electrical conductivity of the samples, the admittance spectra, Y(ω), as a function of frequency, have been analyzed (Equation (4)). The real part of the admittance, Y′(ω), is proportional to the imaginary part of permittivity, ε″(ω), (dielectric loss, Equation (4), which in turn is proportional to the conductivity, σ(ω) [47]. Equations (3) and (4) hold for the real and imaginary parts of the complex permittivity, ε, respectively.

(3) $$\varepsilon^{\prime }\left(\omega \right)=\frac{d}{\omega \epsilon_{0}A}\left(\frac{-Z^{\prime \prime }\left(\omega \right)}{{\left|Z^{*}\right|}^{2}}\right)$$

(4) $$\varepsilon^{\prime \prime }\left(\omega \right)=\frac{d}{\omega \epsilon_{0}A}\left(\frac{Z^{\prime }\left(\omega \right)}{|Z^{*}{|}^{2}}\right)=\frac{d}{\omega \epsilon_{0}A}Y^{\prime }\left(\omega \right)=\frac{1}{\omega \epsilon_{0}}\sigma \left(\omega \right)$$

where *ε′*, and *ε″* denote the real and imaginary part, respectively, *d* the sample thickness (i.e., the distance between the electrodes, different for each applied load), *A* the area (here the one of the top electrode, since it is the smallest), ε_0_ the vacuum permittivity, Z^*^ the complex impedance, Y* = *1*/Z* the complex admittance, ω = 2π*f* the angular frequency, and *σ* the conductivity.

From the admittance spectra, we calculated the frequency-dependent conductivity by applying the following equation [47]:

(5) $$\sigma \left(\omega \right)=\frac{d}{A}Y^{\prime }\left(\omega \right)$$

At low frequencies, the value is associated with the DC conductivity.

The electrical behavior of disc-shaped hydrogel samples was analyzed under various mechanical compressions. The simultaneously measured strain–stress behavior was evaluated by plotting the compressive stress (pressure) versus the compressive strain. We determined the Young modulus of the investigated hydrogels from the linear range of the strain–stress data by linear regression.

In order to verify the behavior of the electrical resistance under repeated mechanical compression, hydrogel samples were squeezed between circular parallel plates of the rheometer, using copper tape on both faces of the sample for electrical contact and measurement of the electrical resistance with an ohmmeter. The initial height of each sample was determined from the gap height between the two geometries by applying a force of 1 N on the sample by the upper part of the rheometer. The samples have been put repeatedly under various mechanical compression by reducing the gap height to achieve 0, 10, 20, 30, and 40% compression. Each resistance measurement has been recorded after 30 s of stabilization. The DC conductivity has been determined by the following equation:

(6) $$\sigma =\frac{1}{\rho }=R\;\frac{A}{L}$$

where σ is the conductivity (S/m), ρ is the resistivity (Ω.m), *R* is the measured resistance (Ω), *A* is the section of the sample (m^2^), and *L* is the thickness of the sample (m).

## 3. Results and Discussion

From an optical inspection, a low mixing (300 rpm) leads to dense hydrogels, while mixing at high speed (1000 rpm and above) leads to hydrogel foams. Gels without PEDOT:PSS appear transparent, hydrogel foams are opaque, and PEDOT:PSS containing hydrogels appear dark bluish to black. All hydrogels containing PEDOT:PSS are electrically conducting. In this section, the quantitative results obtained for the studied hydrogels are presented. The shown data give representative examples of the obtained result.

### 3.1. The Influence of the Mixing Speed and Surfactants on Hydrogel Gels and Foams

**Figure 2:**
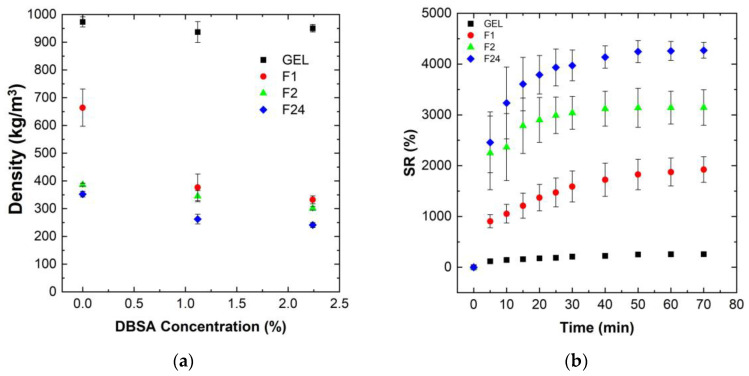
Density and swelling ratio (SR) of PVA/PEDOT:PSS hydrogels with 1.12wt% DBSA: gel (GEL) and foams (F1, F2, F24) prepared at several mixing speeds ranging from 300 rpm to 24,000 rpm (Table 1): (**a**) density as a function of DBSA concentration; (**b**) swelling ratio as a function of time.

Figure 2a shows the influence of the mixing speed, as well as of the DBSA surfactant concentration, on the density of various PVA/PEDOT:PSS hydrogels (gels and foams).

The gels, dense PVA/PEDOT:PSS hydrogels, have a density close to that of water, independent of the DBSA concentration (Figure 2a). This is consistent with the low mixing speed (300 rpm) used for the gel formation, which limits the possible trapping of air bubbles. The DBSA content itself has no significant impact on the hydrogel’s density, while it has a strong influence on reducing the density of the PVA/PEDOT:PSS hydrogel foams (F1, F2, F24) (Figure 2a).

Moreover, for a given DBSA concentration of 1.12wt% or 2.24wt%, the density of the PVA/PEDOT:PSS hydrogels decreases with the increase in mixing speed. The concentration of DBSA used in the hydrogel is beyond the critical micellar concentration in order to have a sufficient quantity of surfactant to stabilize the foam [48]. In fact, to obtain a durable dispersion of a gas in a liquid (i.e., a foam), a surfactant is needed which adsorbs at the gas/liquid interfaces. The structure of stable bubbles results from the adsorption of the surfactant on the film of water, constituting a lamellar wall around the gas bubble [49]. Thus, air bubbles are stabilized by DBSA surfactant molecules, leading to porous hydrogel foams.

Hydrogel foams without DBSA also exhibit a lower density than hydrogel gels, although are less pronounced than for hydrogel foams with DBSA. Similarly, higher mixing speeds lead to lower densities, consistent with the increased trapping of air-bubbles.

Note that as GA was mixed at 1000 rpm for 30 s, air bubbles can thus also be trapped during GA mixing. GA could potentially act as a trapping agent for air bubbles during the reticulation process. However, for hydrogels without DBSA, the coalescence of air bubbles is clearly observed when preparing the PVA/PEDOT:PSS hydrogels, e.g., at 1000 rpm: prior to the completion of the reticulation process, the hydrogel is composed of a gel-structure accumulating at the bottom of the syringes and a foam-structure accumulating at the upper part. Therefore, GA does not efficiently act for trapping air bubbles. The measured densities of samples without DBSA represent average values of non-homogeneous foam-gel structures, which explains the higher density values than observed for the hydrogel foams with DBSA prepared at the same mixing speed. In conclusion, a DBSA content of 1.12wt% significantly stabilizes the obtained foam structure, leading to homogeneous porous hydrogel foams for the investigated mixing speeds (1000 to 24,000 rpm). Thus, the further analysis of water uptake, rheological, electrical and mechanical properties have been focused on homogeneous hydrogels (gels and foams) prepared with 1.12wt% DBSA concentration.

### 3.2. Water Uptake: Swelling Ratio (SR)

The swelling ratio of hydrogels is used to determine the water uptake capacity of the hydrogel structure. Figure 2b shows the measured swelling ratio of PVA/PEDOT:PSS freeze-dried hydrogels as a function of time.

The data show that the swelling ratio, SR, increases rapidly for all foam samples during the first 30 min, and then slowly stabilizes to reach the equilibrium state around 70 min (Figure 2b). The absolute values of the swelling ratio strongly vary between the different hydrogels. Hydrogel foams prepared with the highest mixing speed, which exhibit the lowest initial density in their humid phase, and thus a higher content of incorporated air, also show the highest water uptake performance after freeze-drying.

The incorporation of air into the hydrogels clearly improves the water uptake capacity of the hydrogels. The measured swelling ratios are around 250, 1900, 3140, and 3560% for the PVA/PEDOT:PSS hydrogels prepared at mixing speeds of 300, 1000, 2000, and 24,000 rpm, respectively. These results demonstrate that the incorporation of air-bubbles significantly favors the water uptake performance of the PVA/PEDOT:PSS hydrogel foams. Furthermore, it can be noted that the relative increase in swelling rate from a mixing speed of 1000 rpm to 2000 rpm is significantly higher than that of 2000 rpm to 24,000 rpm.

These findings hold similarly, both for the hydrogels prepared without DBSA and for hydrogels with a DBSA content of 2.24wt%.

**Figure 3:**
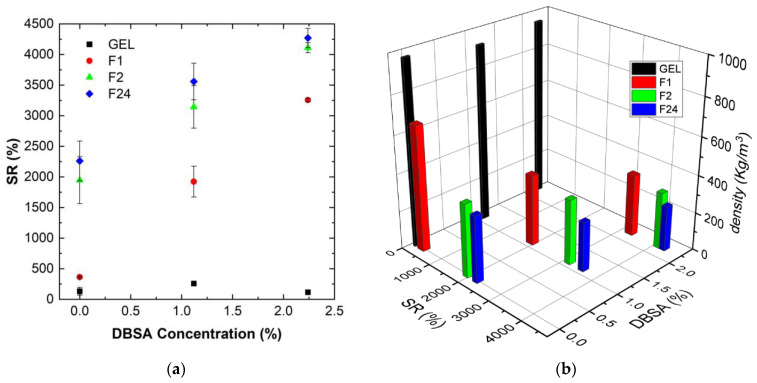
Influence of the DBSA concentration for various PVA/PEDOT:PSS hydrogels (gel and foams (F1, F2, F24)) prepared at various mixing speeds, ranging from 300 rpm to 24,000 rpm (Table 1): (**a**) Swelling ratios (saturated values) for different DBSA concentrations; (**b**) relation between swelling ratio and density for different DBSA concentrations.

Figure 3a shows the measured swelling ratio (upon 70 min of water uptake) for PVA/PEDOT:PSS hydrogels as a function of DBSA concentration. Figure 3b shows the relation between the swelling ratio, the relative DBSA concentration, and the initial density of the humid PVA/PEDOT:PSS hydrogels.

Hydrogels obtained at high mixing speeds and high DBSA contents exhibit higher SR values than hydrogels prepared at low speeds without DBSA (Figure 3a). Moreover, Figure 3b clearly links the water uptake capacity of hydrogels to their structure: hydrogels prepared with a high mixing speed present a low density due to the important incorporation of air. These hydrogels have then a high absorption capacity.

The water uptake capacity of hydrogels prepared at a mixing speed of 24,000 rpm is about 7.5–15 times higher than for hydrogels prepared by magnetic stirring at 300 rpm.

Hydrogels with a low density have an important porosity to absorb solution. It can then be assumed that the high water uptake capacity of foams is favored by a less dense polymer network compared to the gel. This lower network density combined with a high porosity facilitates the foam deformation, which is consistent with the observed higher swelling ratios.

### 3.3. Morphology

**Figure 4:**
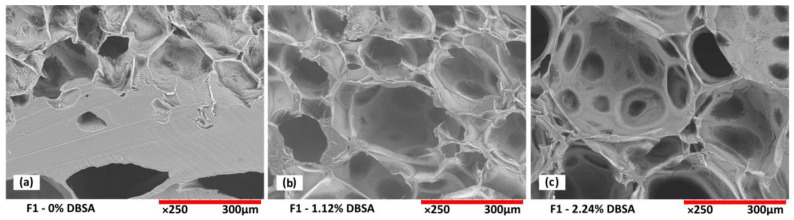
Scanning tunneling microscopy images of PVA/PEDOT:PSS hydrogel foams (F1 prepared at 1000 rpm, Table 1) as a function of DBSA content: (**a**) without DBSA: inhomogeneous foam structure with regions of dense gel formation; (**b**) 1.12wt% DBSA and (**c**) 2.24wt% DBSA: surfactant-stabilized porous structures.

**Figure 5:**
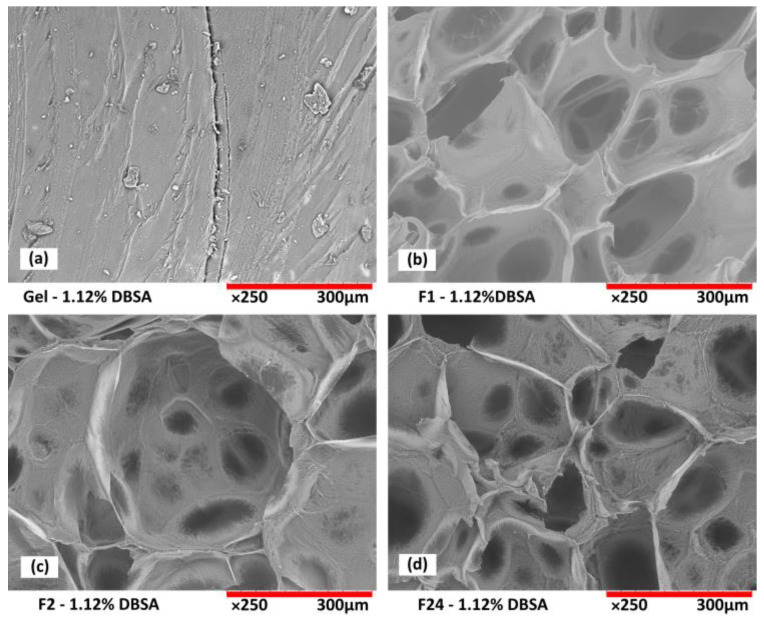
Scanning tunneling microscopy images of PVA/PEDOT:PSS hydrogels as function of the mixing speed. (**a**) Dense gel; (**b**–**d**) foams F1, F2, F24 exhibiting micropores stabilized by DBSA (1.12wt%).

Scanning electron microscopy analysis from the cross-section of freeze-dried hydrogels confirms the porous structure of the foams as a function of DBSA content (Figure 4), and as a function of mixing speed (Figure 5).

For hydrogels without DBSA (Figure 4a), the foam is not stabilized, which leads to the collapse of air bubbles before the end of the reticulation process. Therefore, such hydrogels exhibit an intermittent structure composed of areas of a dense gel phase, mostly present at the bottom of the sample, and areas with a porous structure, preserved by the reticulation process. For hydrogels prepared with DBSA, stable porous structures are obtained (Figure 4b,c). A DBSA content of 1.12wt% leads to a relatively homogenous pore size (Figure 4b), while a DBSA content of 2.24wt% leads to more heterogenous pore sizes.

The number of stabilized air bubbles is higher for increased surfactant concentration, leading to a more porous structure (Figure 4c), which is consistent with a lower density and higher swelling property (Figure 2a and Figure 3).

The influence of mixing speed is also an important parameter to generate different hydrogel morphologies. At a low mixing speed, even with surfactant, no significant incorporation of air is observed. After the freeze-drying process, gel samples present a regular dense structure without significant porosity (Figure 5a).

The increase in the mixing speed leads to a more porous hydrogel structure (Figure 5b–d). At 24,000 rpm, the size of the macroporous structure seems smaller than those obtained at 1000 and 2000 rpm. In addition, the hydrogel presents a microporous structure on the walls of the pores that is not obtained with the lower mixing speeds. This confirms that mixing at high speed induces air incorporation and allows for the fabrication of porous hydrogel foams. Adjusting the air incorporation by the mixing speed allows us to adjust the overall morphology, density, and swelling behavior of the resulting hydrogels.

### 3.4. Gelation Time: Rheology (G′, G″)

**Figure 6:**
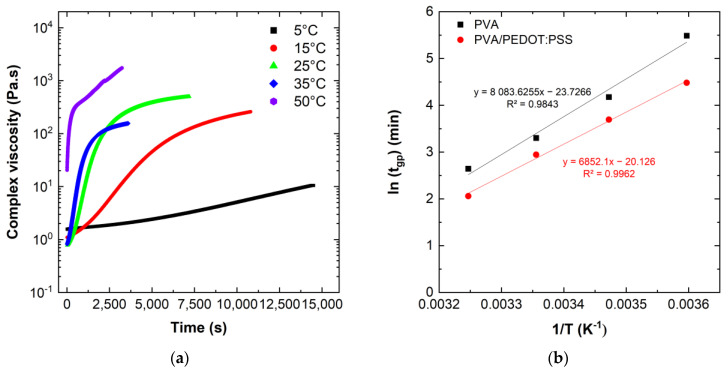
(**a**) Evolution of the complex viscosity versus time during the gelation process for PVA/DBSA hydrogel. (**b**)Temperature dependence of the gelation time for PVA hydrogels and PVA/PEDOT:PSS hydrogels, respectively (1.12wt% DBSA).

Determining the gelation time provides interesting information on the influence of the different constituents forming the hydrogel. As the gelation time of the foams was very fast (less than 5 min at 25 °C), the evolution of the gelation time was only studied for the gels. In order to analyze the influence of PEDOT:PSS on the gel formation, the gelation time of gels composed of DBSA/PVA with or without PEDOT:PSS has been measured for various temperatures between 5 °C and 50 °C. GA has always been added as crosslinker agent. Figure 6a summarize the complex viscosity of PVA/DBSA hydrogels measured during the gelation process, while Table 2 summarizes the measured gelation time of the two different hydrogels at different temperatures.

The evolution of the complex viscosity of the hydrogels with the temperature (Figure 6a) show that an increase in the gelation temperature allows a faster increase in the viscosity. At 5 °C, the complex viscosity increases linearly with a low slope, while at higher temperatures, the complex viscosity increases drastically until it reaches a pseudo-plateau. A high viscosity confers to the hydrogel a more solid state, and the increase in the gelation temperature allows the hydrogels to reach more rapidly this solid state, as mentioned in Table 2. The data in Table 2 show that the PEDOT:PSS containing hydrogels have a lower gelation time. We note that at 50 °C the gelation time of the hydrogels is less than 5 min, which was faster than the required handling of samples to start the rheology measurements, while at 5 °C hydrogels without PEDOT:PSS have a gelation time that exceeds 4 h.

The data show a strong dependence on temperature for the gelation time. Figure 6b shows the gelation point as a function of inverse temperature, 1/T, on a semi-logarithmic plot.

The temperature dependence of the gelation point of PVA and PVA/PEDOT:PSS hydrogels shows indeed an Arrhenius behavior of thermal activation. From the observed Arrhenius relationship (Figure 6b), it can be concluded that the underlying gelation mechanism between PVA and GA is not affected by the change in reaction temperature.

The energy barrier of gelation, also called apparent activation energy, *E_a_*, has been calculated according to the Arrhenius Equation (6) [50]:

(7) $$\ln\left(t_{gp}\right)=C+\frac{E_{a}}{RT}$$

where *t_gp_* is the determined gelation time, *R* the gas constant (8.314 J.K^−1^.mol^−1^), *T* the absolute temperature in Kelvin, and *C* a constant.

The apparent activation energy, *E_a_*, can be calculated from the linear regressions of the Arrhenius plot (Figure 6b). The apparent activation energies of PVA and PVA/PEDOT:PSS hydrogels are 67 ± 6 kJ/mol and 57 ± 2 kJ/mol, respectively, which is in the range of previous studies using GA as crosslinker of PVA hydrogels and lower than for Guar-Gum/GA reaction [50,51].

**Figure 7:**
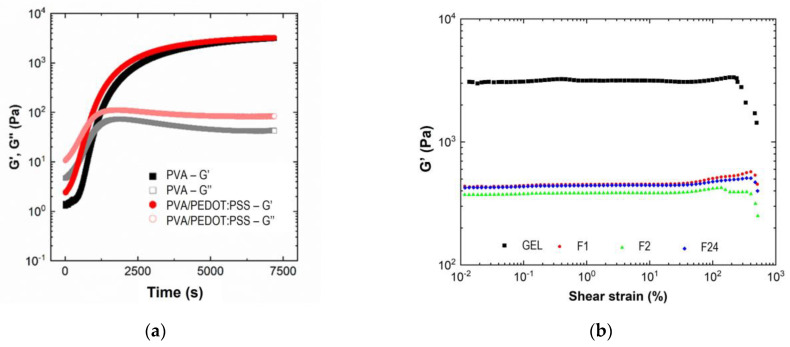
Gelation of hydrogels and viscoelastic properties under shear strain: (**a**) evolution of G′ and G″ as a function of time at 25 °C for PVA- and PVA/PEDOT:PSS hydrogels (GEL, 1.12%wt DBSA); (**b**) variation of the elastic modulus (G′) with shear strain at a fixed frequency, *f* = 1.0 Hz.

These results show that the activation barrier for the gelation of PVA/PEDOT:PSS hydrogels is lower than of PVA hydrogels without the conducting polymer. This can be explained by the interaction between DBSA and PEDOT:PSS in solution before reticulation. As a reminder, PEDOT:PSS is a conducting polymer with a core–shell structure, where the hydrophobic PEDOT^+^ oligomers are surrounded by the hydrophilic and coiled PSS^−^ chains, forming a complex, linked by electrostatic interactions [52]. The PEDOT:PSS core–shell structures can thus be considered complexes suspended in water [53,54]. Moreover, DBSA is known as an anionic surfactant which can form well-defined anionic micellar structures in water when the DBSA content is above the critical micelle concentration. The presence of a sufficient amount of DBSA increases the ionic strength in the hydrogel, which allows us to weaken the electrostatic interactions between PEDOT^+^ and PSS^−^ chains. Thus, the core–shell structure of PEDOT:PSS is modified, and the PEDOT^+^ oligomers are more easily exposed. This facilitates novel intermolecular interactions in which PEDOT^+^ oligomers can both interact with anionic DBSA micelles through electrostatic interactions or organize themselves differently to form a 3D structure due to π–π stacking and hydrophobic attractive interactions [14]. The physically cross-linked DBSA/PEDOT:PSS hydrogel thus creates a second 3D network inside the PVA/GA network. The formation of this double interpenetrating network within the PVA/PEDOT:PSS hydrogel contributes to a reduction in the gelation time determined by the crossing between the storage modulus (G′) and loss modulus (G′) curves. (Figure 7a). Nyström et al. [55], demonstrated that the gelation time was also correlated to the amount of GA present in the mixture. The gelation time observed at 25 °C (Table 2), for a low GA content in the mixture, was quite long and in agreement with their study.

The gelation time of the foam-type hydrogel has been characterized by following the same procedure. For these three types of hydrogels, G′ is always higher than G″, which indicates that the gelation time is less than 5 min, and the rigidity of these hydrogels is quickly obtained.

The hydrogels were further exposed to an increasing oscillation strain at a constant frequency of 1.0 Hz to quantify the linear viscoelastic region (LVR). The LVR is determined as a plateau where the G′ value does not change. The end of the LVR is determined when G′ values are by deviated more than 5% of the average value of the plateau. At this point, the critical deformation of the sample can be determined, i.e., the value of the deformation limit, beyond which the network does not return to its original structure [56]. Figure 7b presents the evolution of G′ and G″ versus shear strain at 25 °C.

The elastic modulus was constant in a long range of shear strain until 250% and 400% for hydrogel in gel and foam state, respectively. This result indicates a better elasticity of the foam-type hydrogel structure. Thus, the structure exhibits a higher stretchability, allowing for the improvement of the swelling properties. Above the LVR, G′ dropped dramatically while G″ increased, which indicates a sudden deformation of the crosslinked network. The rigidity of the gel is 10 times higher than for the foams with G′ values around 3000 Pa and 400 Pa for gels and foams, respectively.

### 3.5. Electrical and Mechanical Properties

The hydrogel’s electrical conductivity was analyzed using broadband dielectric spectroscopy.

**Figure 8:**
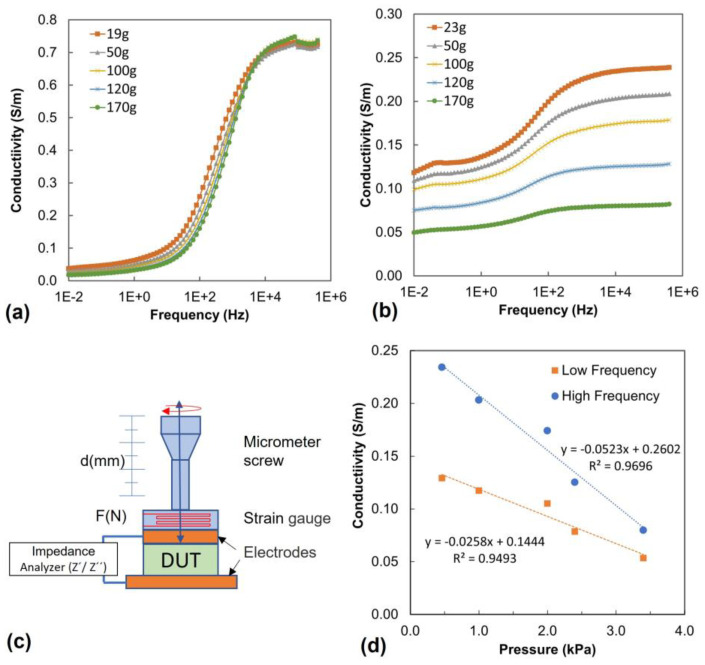
Conductivity of PVA/PEDOT:PSS hydrogels with 1.12wt% DBSA: (**a**) gel, (**b**) foam F2 (mixing speed 2000 rpm), (**c**) scheme of the experimental set-up (d) conductivity as a function of pressure as analyzed from (**b**) at 0.1 Hz (low frequency) and at 1.0 × 10^5^ Hz (high frequency).

Figure 8 shows of conductivity spectra measured under mechanical load for PVA/PEDOT:PSS hydrogels with 1.12wt% DBSA. Figure 8a,b show the conductivity spectra obtained for gel and foam, respectively.

All measured samples exhibit conductivity spectra with two plateaus, one at low frequency and a second one at high frequency. The plateaus can be attributed to the random diffusion of charge carriers, while the region of the frequency-dependent increase in conductivity indicates an activation of charge carriers [57]. In the PVA/PEDOT:PSS hydrogel, the conductivity can originate from ions moving in the liquid or ion hopping along the network, and furthermore, the conductivity of PEDOT:PSS is involved. We note that freeze-dried hydrogel foams with PEDOT:PSS exhibit a significantly lower conductivity in the dry state; we thus assume that the main contribution to the observed conductivity spectra are related to ion diffusion and ion hopping along the hydrogel network.

While the gel shows little dependence for its conductivity on pressure (Figure 8a), the conductivity of foam samples decreases under mechanical load (Figure 8b,d). This behavior is consistent with the mechanical deformation of pores under pressure, which is likely to reduce the available free paths of ions diffusing in the liquid through the porous structure.

In order to quantify the pressure sensitivity in view of sensing applications, we thus analyzed the foam conductivity as a function of pressure, both in the low and high frequency range (Figure 8d). The resulting gauge factors, GF, are at 0.1 Hz (low frequency) GF_LF_ = −26± 3 (S/m)/MPa, and at 1.0·10^5^ Hz (high frequency) GF_HF_ = −52 ± 5 (S/m)/MPa.

As a general trend, fresh PVA/PEDOT:PSS hydrogel foams have a higher conductivity in comparison to rehydrated freeze-dried hydrogels, as well as in comparisons to PVA hydrogel foams without PEDOT:PSS.

**Figure 9:**
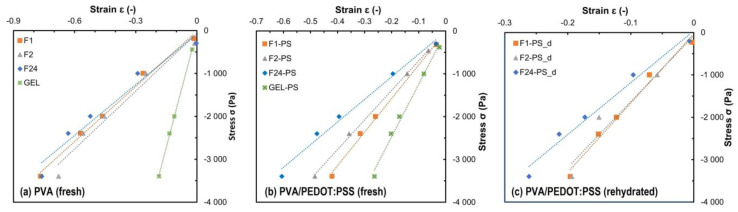
Stress–strain plots of hydrogel (gel and foams with 1.12wt% DBSA) prepared at various mixing speeds ranging from 300 rpm to 24,000 rpm (Table 1) with linear regressions for the analysis of the Young moduli (Table 3): (**a**) PVA hydrogels, (**b**) PVA/PEDOT:PSS hydrogels, (**c**) freeze-dried rehydrated PVA/PEDOT:PSS hydrogels.

Figure 9 shows the linear stress–strain behavior of the hydrogels, allowing us to determine their Young moduli by linear regression. The Young moduli are summarized in Table 3. The freeze-dried hydrogels, as well as gel type hydrogels, exhibit Young moduli above 10 kPa, while the other hydrogels (fresh with and without PEDOT:PSS) show Young moduli below 10 kPa (Table 3). The freeze-drying of the hydrogel foams thus leads to a decrease in the elastic properties; the Young moduli of the foams increase by about a factor of 2.0–2.5, resulting in values equal or up to 33% higher than the Young modulus of the fresh PVA/PEDOT:PSS gel.

**Figure 10:**
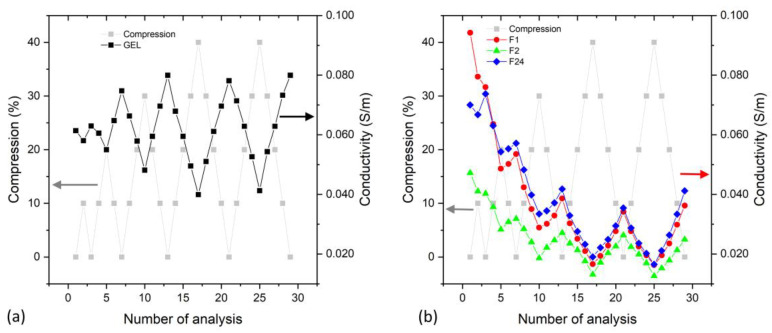
Conductivity measurements performed at different mechanical compressions for (**a**) PVA/PEDOT:PSS hydrogels prepared at 300 rpm and (**b**) PVA/PEDOT:PSS hydrogels prepared from 1000 rpm to 24,000 rpm (1.12wt% DBSA).

In order to evaluate the evolution of the electrical conductivity under repeated compression, the DC conductivity was measured under repeated mechanical compressions (up to 40%). Figure 10 compares the conductivity behavior for PVA/PEDOT:PSS gel (Figure 10a) and PVA/PEDOT:PSS foams (Figure 10b).

The evolution of the conductivity, when the hydrogels are investigated under repeated various mechanical compressions (increased up to 40%, Figure 10), shows that the conductivity of the hydrogels (gel and foams) decreases under compression, which is in agreement with the results of the dielectric spectroscopy analysis (Figure 8). Figure 10a shows that the gel type hydrogel regains its maximum conductivity upon mechanical cycling of up to 40% compression. The observation that the gel’s conductivity increases during the first compressive cycles needs further investigation to understand whether it is related to an improved contact to the electrodes or a pressure-mediated establishment of conductive paths within the hydrogel.

For hydrogel foams, Figure 10b shows a decrease in conductivity during the first compression cycles. We note that compression may squeeze part of the liquid towards the circumference of the hydrogel sample; however, we observed that the hydrogel structures essentially retain their water under repeated compression and release. The strong mechanical compression of the hydrogel foams may also modify their structure and partly degrade the polymeric network or morphology with an impact on the conductivity. Nevertheless, the hydrogel foams show repeatable significant conductivity changes when cycling to the same compression of 40%, which confirms the potential of PVA/PEDOT:PSS hydrogel foams for compressive strain-sensing applications.

## 4. Conclusions

In summary, our investigations have been focused on the development and characterization of PVA/PEDOT:PSS hydrogels, specifically emphasizing the impacts of mixing speed and surfactants on their properties. Our findings exhibit an understanding of hydrogel formation and performance across various conditions. Low mixing speeds (300 rpm) resulted in dense hydrogels, whereas higher speeds (1000 rpm and above) yielded hydrogel foams. Notably, the addition of the DBSA surfactant played a central role, inducing a stable porous structure in hydrogel foams. Gelation time analysis elucidated the rapid gelation of hydrogels, especially for those containing PEDOT:PSS. The synergy between DBSA, PEDOT:PSS, and PVA accelerated the gelation process through the formation of a double polymer network.

From electrical characterizations as a function of frequency, we found that (i) PVA/PEDOT:PSS hydrogels (both gels and foams) exhibit two conductivity plateaus, in the low and high frequency range, respectively, which are likely related to the ionic conductivity of the conductive hydrogels. (ii) The conductivity of the hydrogel foams decreases significantly under compressive load, compared to that of gels. This observation is consistent with the deformation of pores under compression, limiting the ion conductivity inside the porous structure. (iii) Hydrogel foams exhibit linear changes in conductivity as a function of the applied pressure. This makes the hydrogel foams highly interesting in view of compressive strain-sensing applications. Further investigations are needed to elucidate the involved conduction mechanisms in the low and high frequency range in more detail.

## Figures and Tables

**Table 1:** Conditioning of hydrogels (gels and foams) for electrical and mechanical analysis.

Conductive Additive and Sample Status	GEL Mixing Speed (rpm)	Foams F1, F2, F24 Mixing Speed (rpm)
without PEDOT:PSS—fresh	300	1000; 2000; 24,000
with PEDOT:PSS—fresh	300	1000; 2000; 24,000
with PEDOT:PSS—rehydrated	n.a. ^1^	1000; 2000; 24,000

^1^ Freeze-drying of gel samples with PEDOT:PSS leads to mechanically fragile samples, tending to break apart into powder; therefore, rehydrated gel samples were not analyzed.

**Table 2:** Gelation time in seconds, for PVA and PVA/PEDOT:PSS gel-type hydrogels at different temperatures.

Hydrogel’s Gelation Time (s)	5 °C	15 °C	25 °C	35 °C	55 °C
PVA + DBSA	14,500	3910	1630	845	<300
PVA/PEDOT:PSS + DBSA	5295	2412	1140	470	<300

**Table 3:** Young Moduli, *E* (in kPa), for PVA- and PVA/PEDOT:PSS hydrogels (1.12wt% DBSA).

Hydrogels	PVA (Fresh)	PVA/PEDOT:PSS (Fresh)	PVA/PEDOT:PSS (Rehydrated)
GEL	14.0 ± 0.3	12.2 ± 0.7	(-) ^1^
foam F1	4.3 ± 0.2	7.8 ± 0.4	16.3 ± 1.0
foam F2	4.5 ± 0.5	6.9 ± 0.2	15.5 ± 2.4
foam F24	3.9 ± 0.5	5.3 ± 0.3	12.2 ± 1.1

^1^ Freeze-drying of gel samples with PEDOT:PSS leads to mechanically fragile samples; therefore, rehydrated gel samples were not analyzed (Table 1).

## Data Availability

The data presented in this study are available in the article.
